# Supplementary material for: Treatment of Anti-HLA Donor-Specific Antibodies Results in Increased Infectious Complications and Impairs Survival after Liver Transplantation
Source: J Clin Med. 2020 Dec 9;9(12):3986. doi: 10.3390/jcm9123986 (PMC7763868; doi:10.3390/jcm9123986)
Supplement: Supplementary file 1 [file jcm-09-03986-s001.pdf]

Journal of Clinical Medicine

Treatment of Anti-HLA Donor-Specific Antibodies Results in Increased Infectious Complications and Impairs Survival after Liver Transplantation

Sinem Ünlü [1,2], Nils Lachmann [2], Maximilian Jara [1], Paul Viktor Ritschl [1,3], Leke Wiering [1], Dennis Eurich [1], Christian Denecke [1], Matthias Biebl [1], Sascha Chopra [1], Safak Gül-Klein [1], Wenzel Schöning [1], Moritz Schmelzle [1], Petra Reinke [4], Frank Tacke [5], Johann Pratschke [1], Robert Öllinger [1] and Tomasz Dziodzio [1,*]

SUPPLEMENTARY TABLES

Table S1. DSA characteristics of 113 DSA-positive patients who underwent LT from deceased donors between 1 January 2008 and 31 December 2015.

	Total (*n* = 113)		preformed DSA (*n* = 49)		de novo DSA (*n* = 55)		*p*-value [a]	both preformed and de novo DSA (*n* = 9)		*p*-value [b]	*p*-value [c]
**DSA class**											
I	47	(41.6)	32	(65.3)	14	(25.5)	**<0.001**	0	(0.0)	**<0.001**	0.087
II	35	(31.0)	5	(10.2)	28	(50.9)	**<0.001**	2	(22.2)	0.309	0.110
I and II	31	(27.4)	12	(24.5)	13	(23.6)	0.919	7	(77.8)	**0.002**	**0.001**
**DSA count**	1	(1–3)	1	(1–3)	1	(1–2)	0.555	4	(3–5)	**<0.001**	**<0.001**
I	1	(0–2)	1	(1–2)	1	(0–1)	**<0.001**	2	(1–3)	0.577	**0.029**
II	1	(0–1)	0	(0–1)	1	(0–1)	**<0.001**	2	(1–4)	**<0.001**	**0.003**

Annotations: Data presented as *n* (%) or median and Interquartile range (Q1–Q3). [a] Comparison of organ recipients with preformed vs. de novo DSA [b] Comparison with organ recipients with exclusively preformed DSA. [c] Comparison with organ recipients with exclusively de novo DSA. Abbreviations: DSA: Donor-Specific anti-HLA Antibodies, LT: Liver Transplantation.
